# Supplementary material for: Deep learning-assisted genome-wide characterization of massively parallel reporter assays
Source: Nucleic Acids Res. 2022 Nov 9;50(20):11442–54. doi: 10.1093/nar/gkac990 (PMC9723615; doi:10.1093/nar/gkac990)

**Supplemental Results**

**Supplemental Table T1:** Continuation of Figure 2 from the main text. The performance of our model compared with other functional scores over the validation set (20% of confirmed variants matched with background at 1:10 ratio), as measured by area under the ROC curve and area under precision-recall curve. MpraNet (+/bg) indicates the model is trained on MPRA positives and the controlled background set, while MpraNet (+/-) uses the MPRA negatives instead of the background set.

**Supplemental Figure S1:** Comparison of MpraNet model with existing functional scores. a-b: The performance of this model compared with other functional scores over the alternative validation set (positives and negatives from novaSeq and Tewhey), as measured by area under the ROC curve (a) and area under precision-recall curve (b). MpraNet (+/bg) indicates the model is trained on MPRA positives and the controlled background set, while MpraNet (+/-) uses the MPRA negatives instead of the background set.


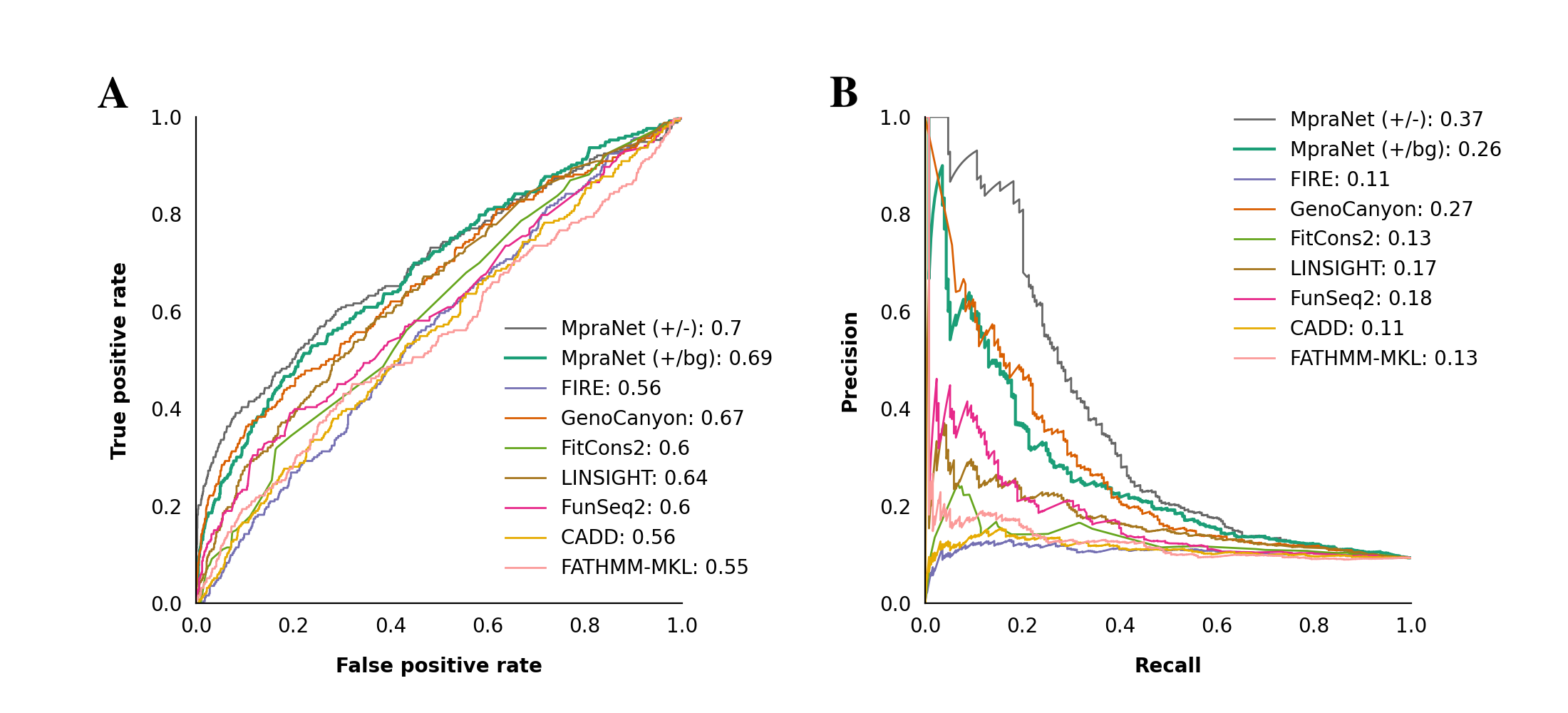


**Supplemental Figure S2:** Comparison of MpraNet with existing functional scores. a-b: The performance of our model compared with other functional scores over the validation set (20% of confirmed variants matched with background at 1:10 ratio), as measured by area under the ROC curve (a) and area under precision-recall curve (b). MpraNet (+/bg) indicates the model is trained on MPRA positives and the controlled background set, while MpraNet (+/-) uses the MPRA negatives instead of the background set.


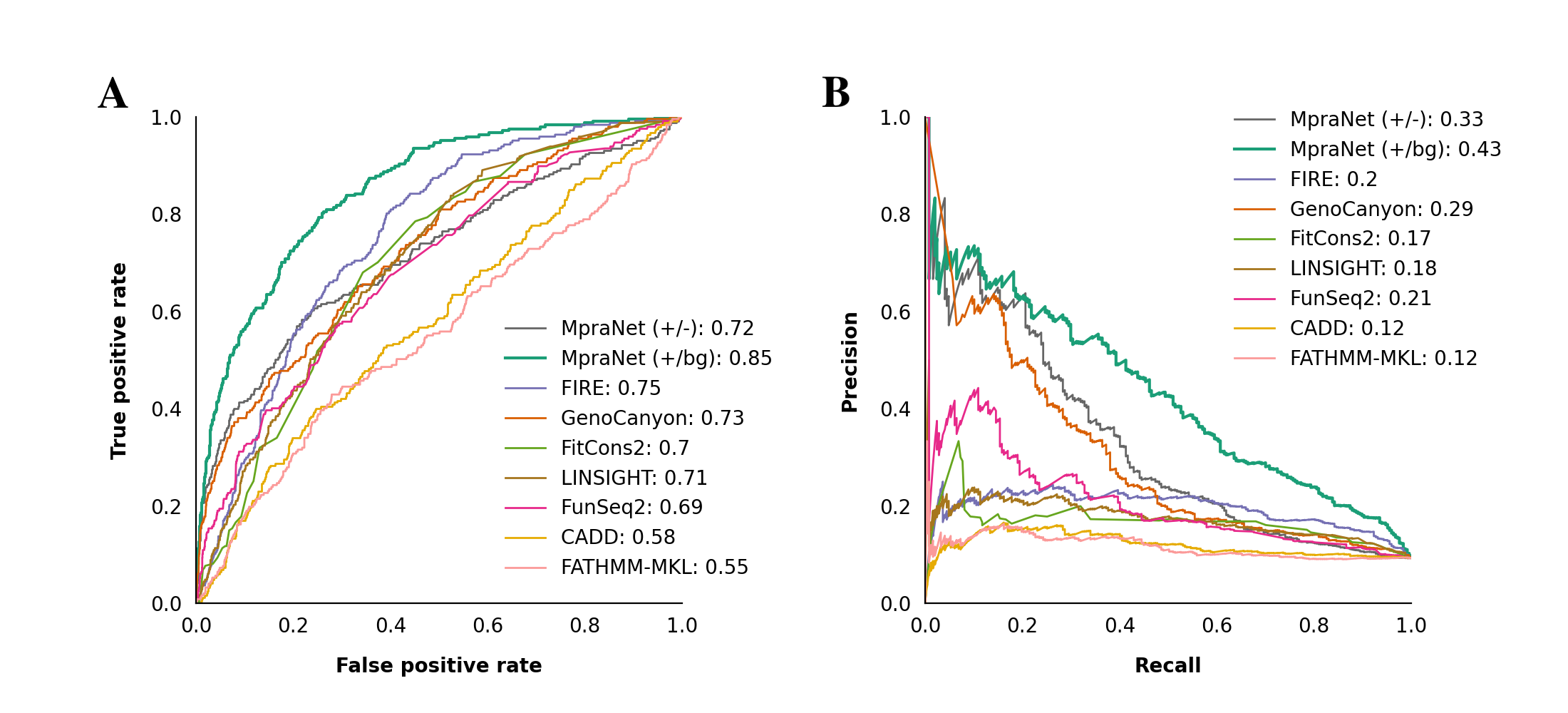


**Supplemental Figure S3**: The functional annotation scores of novaSeq defined positive variants vs. background variants.


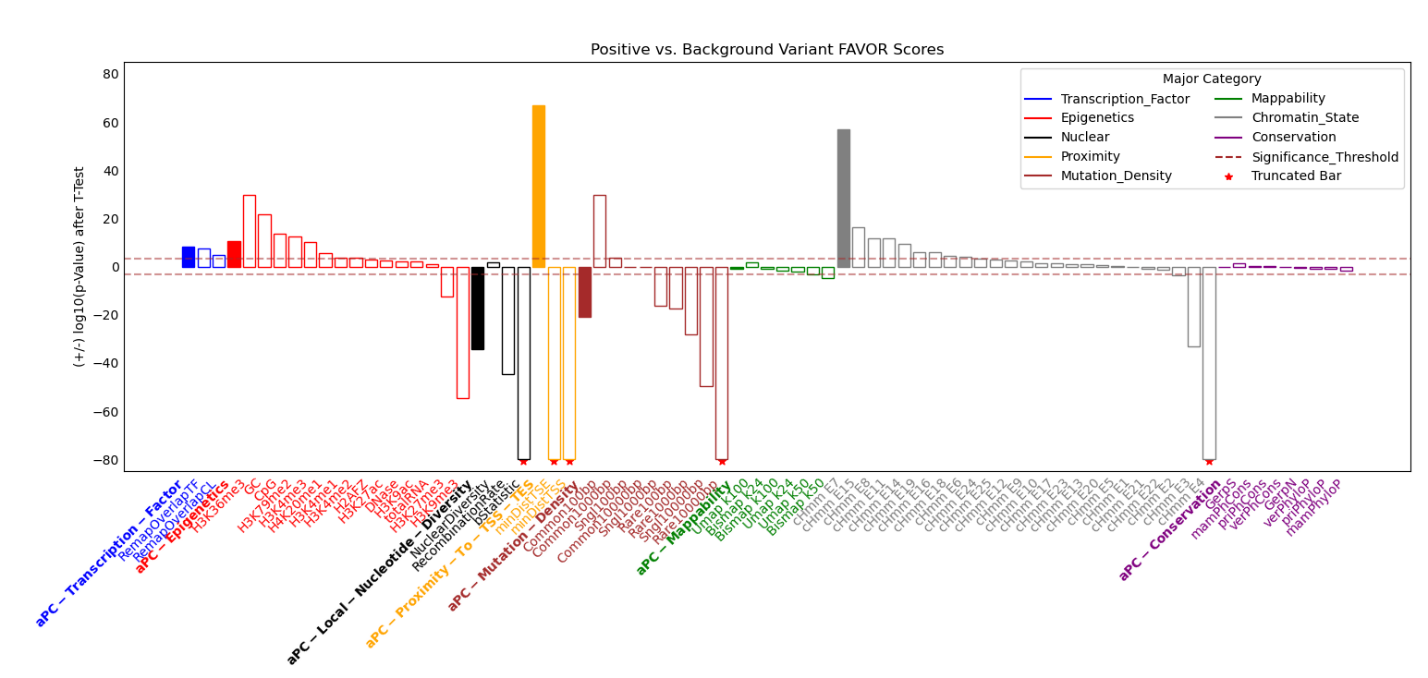

Supplement: gkac990_Supplemental_File [file gkac990_supplemental_file.docx]
